# Empagliflozin-Associated Euglycemic Diabetic Ketoacidosis Masked by Urinary Tract Infection

**DOI:** 10.7759/cureus.66408

**Published:** 2024-08-07

**Authors:** Sukhjinder Chauhan, Victoria Diaz, Ikechukwu R Ogbu, Justin Roy P Sanchez, Andre E Manov, Pinak Shah

**Affiliations:** 1 Internal Medicine, Mountainview Hospital, Las Vegas, USA; 2 Cardiology, Sunrise Health GME Consortium, Las Vegas, USA; 3 Internal Medicine, Medical City Weatherford, Fort Worth, USA; 4 Internal Medicine, Sunrise Health GME Consortium, Las Vegas, USA

**Keywords:** sglt2 eudka, anion gap metabolic acidosis, dka protocol, dka, insulin infusion, eudka, euglycemic ketoacidosis, sglt-2 inhibitors

## Abstract

Sodium-glucose co-transporter 2 (SGLT2) inhibitors have demonstrated efficacy in slowing the progression of chronic kidney disease (CKD), managing conditions such as congestive heart failure (CHF), and reducing cardiovascular and overall mortality in patients with type 2 diabetes mellitus (T2DM). However, their use is associated with complications, including euglycemic diabetic ketoacidosis (euDKA), genital fungal infections, and urinary tract infections (UTIs). Although rare, complications like euDKA can lead to serious consequences if not promptly addressed, as illustrated by this case report of a 90-year-old man with ischemic cardiomyopathy and type 2 diabetes who developed both euDKA and a UTI while on SGLT2 inhibitor therapy. Early identification of euDKA from SGLT2 inhibitor usage prompted cessation of the SGLT2 inhibitor and administration of insulin infusion, ultimately resolving the life-threatening condition.

## Introduction

Euglycemic diabetic ketoacidosis (EuDKA) is a rare complication of sodium-glucose co-transporter 2 (SGLT2) inhibitors that can sometimes go unnoticed in patients, potentially resulting in worse outcomes. SGLT2 inhibitors, which have shown efficacy in improving patient outcomes in conditions including heart failure, diabetes mellitus, and diabetic nephropathy, saw a consistent increase in utilization by cardiologists, endocrinologists, and nephrologists from 2015 to 2021 [1].

DKA, typically associated with Type 1 or Type 2 diabetes mellitus, presents with hyperglycemia, metabolic acidosis, and ketonemia/ketonuria. However, some patients on SGLT2 inhibitors may develop euDKA, characterized by elevated anion gap metabolic acidosis and ketonemia/ketonuria despite blood glucose levels below 250 mg/dL [2-3].

## Case presentation

A 90-year-old male presented to the hospital with a chief complaint of dysuria and abdominal pain associated with fevers and chills over the last three to four days His past medical history included ischemic cardiomyopathy, heart failure with reduced ejection fraction and left ventricular ejection fraction of 25-30%, paroxysmal atrial fibrillation, left and right bundle branch block requiring pacemaker, and multiple myeloma in remission. The patient denied any chest pain, shortness of breath, orthopnea, or paroxysmal nocturnal dyspnea during this admission. The patient reported compliance with his medications: empagliflozin 25 mg, metoprolol tartrate 25 mg twice daily, isosorbide mononitrate 60 mg, and Eliquis 2.5 mg twice daily.

Upon admission, the patient's vitals were stable, with blood pressure ranging from 120s/60s mmHg, saturating 95% oxygen on room air. Physical examination was remarkable for suprapubic tenderness without evidence of costovertebral tenderness. Lung auscultation revealed clear breath sounds, and no edema was observed in the bilateral lower extremities, suggesting no signs of heart failure exacerbation. The chemistry panel in Table 1 was remarkable for a serum bicarbonate level of 17 mEq/L and an elevated albumin-adjusted anion gap of 17 mEq/L. Additionally, the panel showed a blood glucose level of 130 mg/dL, blood urea nitrogen (BUN) of 21 mg/dL, and serum creatinine level of 0.92 mg/dL. A serum lactic acid level was obtained due to the elevated anion gap, which was elevated at 3.0 mmol/L. Urinalysis from Table 2 showed moderate ketonuria and markedly elevated urine glucose of 1,000 mg/dL. The urinalysis also indicated the presence of bacteriuria and leukocyte esterase.

**Table 1:** Results of chemistry metabolic panel (CMP) on admission

Comprehensive metabolic panel	Results (reference)
Sodium	136 mmol/L (135–45)
Potassium	3.9 mmol/L (3.5–5.5)
Chloride	106 mmol/L (93–107)
Bicarbonate (CO_2_)	17 mmol/L (21–32)
Albumin-adjusted anion gap	16 mmol/L (4–12)
Blood urea nitrogen (BUN)	21 mg/dL (7-18)
Creatinine (Cr)	0.92 mg/dL (0.52-1.23)
Albumin	2.8 g/dL (3.4-5.0)
Lactic acid	3.0 mmol/L (0.4-2.0)
Glucose	130 mg/dL (70-110)

**Table 2:** Urinalysis results were positive for moderate ketones

Urinalysis	Results (Normal reference)
Urine Color	Yellow (Yellow)
Urine Appearance	Cloudy (Clear)
Urine pH	5.5 (5.0-9.0)
Urine Protein	100 mg/dL (Negative)
Urine Nitrites	Positive (Negative)
Urine Leukocyte Esterase	Large (Negative)
Urine Glucose	1000 + mg/dL
Urine Ketones	Moderate

The patient's clinical presentation suggested a UTI accompanied by an anion gap metabolic acidosis, possibly secondary to lactic acidosis stemming from UTI-related sepsis or euDKA secondary to empagliflozin, an SGLT2 inhibitor. Notably, empagliflozin was not initiated during hospitalization. Following treatment with intravenous fluids (IV) and antibiotics for the UTI, lactic acidosis resolved. However, the anion gap increased from 16 to 22.8 mmol/L (Table 3) despite the resolution of lactic acidosis. Persistent elevation of anion gap metabolic acidosis prompted testing for beta-hydroxybutyrate, revealing beta-hydroxybutyrate levels of 37.4 mg/dL, and arterial blood gas (ABG) analysis (Table 4) showed a pH of 7.28 with a bicarbonate level of 14 mEq/L and partial pressure of carbon dioxide (PaCO_2_) of 31, consistent with euDKA. The patient reported taking their final dose of empagliflozin just before hospital admission approximately 24 hours ago.

**Table 3:** Results of chemistry metabolic panel (CMP) day after the initial treatment

Comprehensive metabolic panel	Results (reference)
Sodium	138 mmol/L (135–45)
Potassium	4.0 mmol/L (3.5–5.5)
Chloride	105 mmol/L (93–107)
Bicarbonate (CO2)	14 mmol/L (21–32)
Albumin-adjusted anion gap	22.8 mmol/L (4–12)
Blood urea nitrogen (BUN)	17 mg/dL (7-18)
Creatinine (Cr)	0.98 mg/dL (0.52-1.23)
Albumin	2.5 g/dL (3.4-5.0)
Lactic acid	1.2 mmol/L (0.4-2.0)
Glucose	113 mg/dL (70-110)

**Table 4:** Arterial blood gas (ABG) analysis

Arterial Blood Gas (ABG)	Results (reference)
pH	7.28 (7.35-7.45)
PaCO2	31 mmHg (35-40)
HCO3 (bicarbonate)	14 mEq/L (22-24)

Following the diagnosis of euDKA, the patient was promptly transferred to the ICU for initiation of an insulin drip. Over the subsequent 36 hours in intensive care, the patient received 5-6 liters of intravenous fluids. During this time, the anion gap metabolic acidosis resolved, with levels decreasing from 22.8 mg/dL (Table 3) to 11.5 mg/dL (Table 5).

**Table 5:** Chemistry metabolic panel (CMP) results showing resolution of anion gap metabolic acidosis

Comprehensive metabolic panel	Results (reference)
Sodium	138 mmol/L (135–45)
Potassium	3.9 mmol/L (3.5–5.5)
Chloride	109 mmol/L (93–107)
Bicarbonate (CO2)	22 mmol/L (21–32)
Albumin-adjusted anion gap	12 mmol/L (4–12)
Blood urea nitrogen (BUN)	11.5 mg/dL (7-18)
Creatinine (Cr)	0.83 mg/dL (0.52-1.23)
Albumin	2.2 g/dL (3.4-5.0)
Lactic acid	1.8 mmol/L (0.4-2.0)
Glucose	144 mg/dL (70-110)

Following the resolution of the elevated anion gap, the insulin drip was discontinued. The patient was started on a low-carbohydrate diet and started on sliding-scale insulin. On discharge, the SGLT2 inhibitor was discontinued permanently and the patient was recommended to follow up with his primary care physician.

## Discussion

EuDKA manifests with metabolic acidosis (pH < 7.3), bicarbonate levels < 18 mEq/L, and an elevated anion gap (> 12) concomitant with serum glucose concentrations < 250 mg/dL and ketonemia [2-4]. Predisposing risk factors include pregnancy, prolonged periods of fasting, surgical interventions, acute pancreatitis, glycogen storage disorders, chronic hepatic ailments, gastroparesis, and malfunction of insulin pumps. Notably, SGLT2 inhibitors have been also associated with a rare incidence of euDKA [5].

Food and Drug Administration (FDA)-approved SGLT-2 inhibitors, including canagliflozin, dapagliflozin, empagliflozin, and ertugliflozin, exert their blood glucose-lowering effects by inhibiting SGLT-2 transporters in the renal system, thereby enhancing urinary glucose excretion. Their approved indications encompass T2DM accompanied by atherosclerotic cardiovascular disease, hypertension, heart failure with preserved or reduced ejection fraction, and chronic kidney disease such as diabetic nephropathy [6-7]. Studies have shown that SGLT-2 inhibitors have demonstrated efficacy in reducing both systolic and diastolic blood pressure, as evidenced by findings from trials such as EMPA-REG BP [8]. Furthermore, these agents exhibit renal protective properties, effectively slowing the progression of diabetic nephropathy. For instance, in the EMPA-REG OUTCOME study, empagliflozin demonstrated a 46% reduction in the composite renal outcome [9].

SGLT2 inhibitors are linked to several adverse effects, including euDKA, genital mycotic infections, and urinary tract infections. The development of euDKA with these inhibitors is attributed to decreased insulin production, increased glucagon secretion, and subsequent processes such as gluconeogenesis, glycogenolysis, lipolysis, and ketogenesis [10]. Normally, glucose influx into pancreatic alpha cells elevates the adenosine triphosphate (ATP) to adenosine diphosphate (ADP) ratio, reducing potassium efflux and calcium influx, thus decreasing glucagon secretion. However, under SGLT-2 inhibitor influence, glucose influx into these cells decreases, lowering the ATP/ADP ratio, increasing potassium efflux, and elevating calcium influx, leading to heightened glucagon secretion. This results in a higher glucagon/insulin ratio, promoting lipolysis, ketogenesis, and a shift toward fat metabolism, ultimately leading to ketone production [11].

Furthermore, these inhibitors enhance urinary glucose excretion, consequently lowering blood glucose levels, which further diminishes insulin secretion from pancreatic beta cells, leading to excess ketone body production. Additionally, their inhibition of SGLT-2 transporters establishes a positive nephron electrochemical gradient, augmenting renal reabsorption of ketone bodies and elevating serum ketone levels [12-13]. For patients who develop euDKA due to an SGLT-2 inhibitor, management entails promptly discontinuing the medication and initiating the DKA protocol, which includes administering intravenous fluids, electrolytes, and insulin [2,14].

Our patient, on an SGLT2 inhibitor as part of goal-directed therapy for HFrEF, presented with dysuria and was diagnosed with a UTI, prompting the start of antibiotic treatment. During the initial assessment, an elevated anion gap suggested potential causes, including sepsis-related lactic acidosis from the UTI or the SGLT2 inhibitor therapy. Although lactic acidosis resolved with IV fluid resuscitation, high anion gap metabolic acidosis persisted. The persistent elevation of the anion gap metabolic acidosis prompted testing for beta-hydroxybutyrate, which revealed a notably high level of 37.4 mg/dL. ABG analysis (Table 4) showed a pH of 7.28, a bicarbonate level of 14 mEq/L, and a PaCO2 of 31 mmHg, consistent with euDKA, likely attributable to the SGLT2 inhibitor. The patient had taken their final dose of empagliflozin approximately 24 hours before admission. Given the half-life of empagliflozin is about 12.4 hours, it generally takes around 4 half-lives to eliminate over 97% of the medication from the plasma [15]. Following the diagnosis of euDKA, the patient was transferred to the ICU for an insulin drip and received 5-6 liters of intravenous fluids over the next 36 hours. During this period, the anion gap metabolic acidosis resolved, with levels decreasing from 22.8 mg/dL (Table 3) to 11.5 mg/dL (Table 5).

In our patient's case, his anion gap metabolic acidosis persisted for approximately 48 hours (about 2 days) until insulin therapy was initiated. Following the diagnosis of euDKA, the patient was effectively treated with insulin infusion, IV fluids, and electrolyte replacement. The patient's UTI was likely caused by empagliflozin, as it increases glucosuria increases the risk of UTI. Rarely, it can also lead to euDKA, as seen in our patient's case. Consequently, empagliflozin was discontinued indefinitely upon euDKA diagnosis. 

## Conclusions

As SGLT2 inhibitors become more widely used for various conditions, healthcare providers need to be aware of euDKA as a rare side effect of SGLT2 inhibitor therapy. Unlike typical DKA, euDKA may not show high blood sugar levels. This can be confusing because other conditions, like infections, can also cause similar symptoms or elevated anion gap metabolic acidosis. Our case highlights this challenge and diagnostic dilemma. The patient's elevated anion gap could have been mistaken for another issue. To ensure the best outcome, healthcare providers should consider euDKA as a possibility and one of the differentials for patients on SGLT2 inhibitors who have persistent elevated anion gap metabolic acidosis.

## References

[1] Alkabbani W, Gamble JM (2023). Prescribing trends of the sodium-glucose cotransporter-2 inhibitors among different physician specialties in Canada (2015-2021). Can J Diabetes.

[2] Rawla P, Vellipuram AR, Bandaru SS, Pradeep Raj J (2017). Euglycemic diabetic ketoacidosis: a diagnostic and therapeutic dilemma. Endocrinol Diabetes Metab Case Rep.

[3] Evans K (2019). Diabetic ketoacidosis: update on management. Clin Med (Lond).

[4] Peters AL, Buschur EO, Buse JB, Cohan P, Diner JC, Hirsch IB (2015). Euglycemic diabetic ketoacidosis: a potential complication of treatment with sodium-glucose cotransporter 2 inhibition. Diabetes Care.

[5] Nasa P, Chaudhary S, Shrivastava PK, Singh A (2021). Euglycemic diabetic ketoacidosis: a missed diagnosis. World J Diabetes.

[6] Nespoux J, Vallon V (2020). Renal effects of SGLT2 inhibitors: an update. Curr Opin Nephrol Hypertens.

[7] Simes BC, MacGregor GG (2019). Sodium-glucose cotransporter-2 (SGLT2) inhibitors: a clinician’s guide. Diabetes Metab Syndr Obes.

[8] Tikkanen I, Narko K, Zeller C, Green A, Salsali A, Broedl UC, Woerle HJ (2015). Empagliflozin reduces blood pressure in patients with type 2 diabetes and hypertension. Diabetes Care.

[9] Bailey CJ, Day C, Bellary S (2022). Renal protection with SGLT2 inhibitors: effects in acute and chronic kidney disease. Curr Diab Rep.

[10] Dutta S, Kumar T, Singh S, Ambwani S, Charan J, Varthya SB (2022). Euglycemic diabetic ketoacidosis associated with SGLT2 inhibitors: a systematic review and quantitative analysis. J Family Med Prim Care.

[11] Chauhan S, Manov A, Dhillon GS, Shah P (2023). Empagliflozin-associated euglycemic diabetic ketoacidosis in a patient with type 2 diabetes mellitus. Cureus.

[12] Hattersley AT, Thorens B (2015). Type 2 diabetes, SGLT2 inhibitors, and glucose secretion. N Engl J Med.

[13] Gajjar K, Luthra P (2019). Euglycemic diabetic ketoacidosis in the setting of SGLT2 inhibitor use and hypertriglyceridemia: a case report and review of literature. Cureus.

[14] Goldenberg RM, Berard LD, Cheng AY, Gilbert JD, Verma S, Woo VC, Yale JF (2016). SGLT2 inhibitor-associated diabetic ketoacidosis: clinical review and recommendations for prevention and diagnosis. Clin Ther.

[15] Ndefo UA, Anidiobi NO, Basheer E, Eaton AT (2015). Empagliflozin (Jardiance): a novel SGLT2 inhibitor for the treatment of type-2 diabetes. P T.

